# Galactin-3 regulation of CDC42 promotes neuronal autophagy following spinal cord injury

**DOI:** 10.3389/fncel.2025.1622825

**Published:** 2025-10-15

**Authors:** Lei Yan, Xun Zhou, Qianqiu Li, Hongxiang Hong, Chunshuai Wu, Yong-Jing Gao, Zhiming Cui, Guanhua Xu

**Affiliations:** 1 The First People’s Hospital of Nantong, The Second Affiliated Hospital of Nantong University, Research Institute for Spine and Spinal Cord Disease of Nantong University, Jiangsu, China; 2 Institute of Pain Medicine and Special Environmental Medicine, Co-Innovation Center of Neuroregeneration, Nantong University, Jiangsu, China

**Keywords:** spinal cord injury, neuron, autophagy, GAL3, CDC42

## Abstract

**Background:**

Spinal cord injury (SCI) is a debilitating condition within the nervous system with a high disability rate and substantial economic burden. The functional recovery following SCI is enhanced by moderate levels of autophagy but hindered when autophagy becomes excessive. Galectin-3 (GAL3) has been recognized as an autophagy regulator; however, its role in SCI and its associated mechanism are largely unknown.

**Methods:**

The Walsh clamping method was employed to establish a rat SCI model, while a high-concentration glutamate incubation method was used to create an *in vitro* model of spinal cord neuronal injury. Subsequent to establishing the injury models, the expression levels of GAL3 were detected using QPCR and Western Blot. Immunohistochemical staining was performed to determine the localization of GAL3 expression. SiR-GAL3 or GAL3 inhibitors were utilized to knock down or inhibit GAL3 expression, and behavioral analysis was conducted to assess the recovery of motor function in rats following SCI. Bioinformatics analysis was carried out to explore the mechanism of action of GAL3 post-SCI. Western Blot was used to examine the relationship between the expression levels of GAL3 and autophagy-related proteins following SCI. Sequencing analysis was performed to identify the differential gene expression in spinal cord neurons with knocked-down GAL3 compared to the control group after neural injury, aiming to investigate the mechanism of action between GAL3 and its downstream target gene Cell-division-cycle-42 (CDC42). Co-IP was employed to detect the interaction between GAL3 and CDC42 proteins. Western Blot was used to analyze the relationship between CDC42 and autophagy-related protein expression levels following *in vitro* stimulation of neurons with GAL3. Molecular biology experiments were conducted to assess the expression levels and localization of CDC42 post-SCI. Behavioral analysis was performed to evaluate the recovery of motor function in rats with inhibited CDC42 expression after SCI. ELISA was used to measure the expression levels of GAL3 and CDC42 in both rat and human samples post-SCI.

**Results:**

We found that GAL3 was increased in spinal neurons and serum in SCI rats, and knockdown or inhibition of GAL3 promoted motor function recovery. The bioinformatics analysis showed that GAL3 is closely related to programmed cell death after SCI. Indeed, the knockdown of GAL3 resulted in a decrease in autophagy markers ATG7 and LC3 II/I ratio, along with an increase in P62 expression. Furthermore, GAL3 and CDC42 exhibited close associations with neuronal autophagy. Injection of siR-CDC42 and CDC42 inhibitor ML141 effectively reduced GAL3-mediated enhancement of neuronal autophagy. Additionally, CDC42 was increased in spinal neurons post-SCI, and administration of ML141 decreased the expression of autophagy markers and improved motor function recovery. Importantly, elevated levels of GAL3 and CDC42 were observed in the serum of SCI patients.

## 1 Introduction

Spinal cord injury (SCI) is a serious injury of the central nervous system (CNS), with high incidence, notable disability rates, elevated mortality, and substantial economic burdens (Hu et al., 2023). Worldwide, about 700,000 new cases of SCI are reported annually (Kumar et al., 2018), and over 60,000 new cases are documented each year in China (Jiang et al., 2021). Patients may experience temporary or permanent impairment of sensory, motor, and autonomic nerve functions below the level of SCI. These effects can profoundly disrupt their daily life and work, consequently leading to a considerable societal burden (Karsy and Hawryluk, 2019). Despite remarkable advancements in medical care for treating spinal cord injuries, the current treatment programs predominantly focus on providing supportive measures (Calvert et al., 2019; Lavis and Goetz, 2019; Thomaz et al., 2019).

Following SCI, a cascade of complex inflammatory reactions is triggered, leading to secondary injury that results in axonal rupture and neuronal death. The process disrupts neural circuit integrity, ultimately impeding the recovery of spinal cord function (Alizadeh et al., 2019; Hu et al., 2023; Wu and Lipinski, 2019). Autophagy, alongside apoptosis and necrosis, represents one of the primary morphological types of cell death (Lavis and Goetz, 2019). Previous studies have indicated an elevation of autophagy markers such as LC3 and Beclin 1 in various experimental models of SCI (Hao et al., 2013; Kanno et al., 2011; Munoz-Galdeano et al., 2018; Sekiguchi et al., 2012; Tanabe et al., 2011). Although autophagy changes after SCI are different across species, injury models, and injury severity, the majority of data suggest inhibited autophagy flow within the injured spinal cord (Wu and Lipinski, 2019). Notably, neuroautophagy is prominently associated with autophagic dysfunction following traumatic brain injury and SCI (Lipinski et al., 2015). Therefore, it is important to clarify the mechanism of neuronal autophagy following SCI for exploring potential interventions aimed at enhancing functional recovery post-SCI.

Galectin-3 (GAL3) is a β-galactoside-bound cytoplasmic lectin, which regulates various biological functions, including cell proliferation, differentiation, migration, and immune response (Dong et al., 2018). Within the CNS, GAL3 exhibits both pro-inflammatory and anti-inflammatory effects due to different cell types and cell locations (Chaudhary et al., 2022; Soares et al., 2021). For example, in a mouse model of acute peripheral inflammation, GAL deficiency led to the absence of IL-17, IFN-γ, and TNF-α transcripts in the CNS, indicating a reduction in neuroinflammation compared to wild-type mice (Espinosa-Oliva et al., 2021). On the contrary, GAL3 treatment downregulates the inflammatory signaling pathway, exerting a protective effect in conditions like stroke in rats, thereby mitigating apoptosis and neurodegeneration (Wesley et al., 2021). In addition, GAL3 has been considered an autophagy regulator in recent years (Zheng et al., 2023). It participates in collecting damaged endosome/phagocyte membranes and lysosomes in response to various stimuli, emerging as an important tool for detecting membrane damage such as lysosomal injury (Alvarez-Valadez et al., 2021). However, debates persist concerning the precise role of GAL3 in autophagy regulation (Zheng et al., 2023). Our previous dataset showed an increase in GAL3 mRNA level after SCI in rats (Yan et al., 2022). Further investigation is necessary to uncover the role and mechanism of GAL3 in CNS autophagy.

The Ras Homology Family of Guanosine triphosphates (Rho GTPases), such as Ras Homolog Family Member A (RhoA), Rac Family Small GTPase 1 (Rac1), and Cell Division Cycle 42 (CDC42), play a crucial role in modulating various cell functions, including cell morphology, migration, endocytosis, and cell cycle progression (Mosaddeghzadeh and Ahmadian, 2021). Functioning as switches for signal transduction, they alternate between GDP-binding forms and active GTP-binding forms (Walker and Olson, 2005). CDC42 plays an important role in regulating neuronal polarization and axon formation in the developing brain (Garvalov et al., 2007). In addition, Rho GTP enzymes, including CDC42, are unignorable in guiding the injury and regeneration of neurons (Bradke, 2022). Injuries and diseases in the CNS induce the activation of RhoA and its downstream effector Rho kinase (ROCK), thus promoting cell death and retraction and the loss of axons and synapses (Mulherkar and Tolias, 2020). However, the autophagy function of CDC42 in neurons after SCI is still unclear. While studies have reported that elevated levels of CDC42 mRNA in spinal neurons and glial cells of rats after SCI (Erschbamer et al., 2005), the specific mechanism of CDC42 post-SCI remains to be fully clarified.

Our present study demonstrated that the expression of GAL3 and CDC42 protein is increased in neurons following SCI, and GAL3 promotes the expression of autophagy in neurons. Interestingly, inhibiting the expression of GAL3 and CDC42 proteins at the SCI site enhances the recovery of motor function. Our findings reveal novel downstream targets for the modulation of autophagy post-SCI and provide preliminary evidence and a foundation for developing new drugs and therapies aimed at treating SCI.

## 2 Materials and methods

### 2.1 Animals

Healthy adult female Sprague-Dawley rats (180–220 g, 8 weeks old) were obtained from the Animal Center of Nantong University. Animals were housed under standard conditions (12 h light/dark cycle, 23 ± 1°C) with *ad libitum* access to food and water, followed by a 1 week acclimation period. All procedures strictly adhered to the NIH *Guide for the Care and Use of Laboratory Animals* (8th ed., 2010) and were approved by the Animal Ethics Committee of Nantong University (Protocol Nos. S202112294-08 and S20230727-007). Animal welfare was prioritized, with measures implemented to minimize distress.

### 2.2 SCI model

Prior to any surgical procedures that could potentially cause pain or discomfort, animals were administered appropriate anesthesia or analgesia. Following best practices recommended for the species and the nature of the surgery, the SCI model was established in rats through the intraperitoneal administration of avertin (Kuhn and Wrathall, 1998) (2,2,2-tribromoethanol, Sigma-Aldrich, 250 mg/kg) to induce anesthesia. Subsequently, a Walsh impactor weighing 35 g was used to apply compression at the T7–T9 level for 1 min. The successful induction of SCI was confirmed by the observation of hind limb flaccid paralysis. Serum and spinal cord samples were collected on the 3rd, 7th, and 14th day post-SCI. Each group, namely the sole SCI group and the Sham group, consisted of 6 rats.

In the second and third experiments, treatment compounds were administered to rats after SCI. The daily administration of compounds to rats at the lesion site was performed using a Hamilton 701N micropipette. The injection was made percutaneously into the injury site, with the injection point 1 mm from the edge of the lesion and at a depth of 1.5 mm. Starting from the day of the surgical procedure and for the following 7 days, treatment compounds were injected daily into the injured area. During the study, all rats were administered antibiotics for 7 days and underwent artificial bladder drainage twice daily until bladder function was restored. The detailed experimental grouping information was presented in the following table. All treatments in Table 1 utilized the same vehicle solution.

**TABLE 1:** Detailed experimental grouping information.

Experiment	Group	Sample size	Intervention protocols
Experiment 1	Sham	6	Surgical exposure without impactor compression
	SCI-3	6	Euthanized at the 3 days post-injury timepoint for serum and spinal cord tissue collection
	SCI-7	6	Euthanized at the 7 days post-injury timepoint for serum and spinal cord tissue collection
	SCI-14	6	Euthanized at the 14 days post-injury timepoint for serum and spinal cord tissue collection
Experiment 2	Sham + Vehicle	8	Surgical exposure without impactor compression + vehicle solution
	SCI + Vehicle	8	SCI + vehicle solution
	SCI + siR-GAL3	8	SCI + siR-GAL3 (400 nmol/kg, siRNA ID. 195338, Catalog. AM16708, Invitrogen)
	SCI + TD139	8	SCI + TD139 (GAL3 inhibitor, 400 mg/kg, No. T899651g, Macklin)
	SCI + GAL3	8	SCI + GAL3 (400 mg/kg, No. HY-P77684, MedChemExpress)
Experiment 3	Sham + Vehicle	8	Surgical exposure without impactor compression + vehicle solution
	SCI + Vehicle	8	SCI + vehicle solution
	SCI + siR-CDC42	8	SCI + siR-CDC42 (400 nmol/kg, siRNA ID. 196388, Catalog. AM16708, Invitrogen)
	SCI + ML141	8	SCI + ML141 (CDC42 inhibitor, 400 mg/kg, No. HY-12755, MedChemExpress)

In accordance with the AVMA (American Veterinary Medical Association) guidelines for animal euthanasia, CO_2_ asphyxiation was used for sedation and euthanasia when necessary. The experimental animals were placed in a CO_2_ anesthesia chamber, and the CO_2_ valve was opened (65% vol/min). Once the animals gradually lost consciousness, the CO_2_ concentration was increased to 100%, resulting in an unconscious state in the rats. Ventilation was continued for an additional 2 min and cervical dislocation was performed to ensure death. A total of 84 rats were euthanized, and this was performed under the following conditions: upon obtaining experimental results or at the end of the animal experiment (80 rats); when, based on the veterinarian’s assessment considering factors such as body weight, appetite, infection, and dying symptoms, the degree of suffering reached or exceeded the predefined humane endpoint (four rats); and due to other reasons rendering them unsuitable for further housing (0 rats).

### 2.3 Evaluation of motor function recovery of hind limbs in rats

On the 3rd, 7th, 14th, 21st, and 28th day post-SCI, the rats were scored with the Basso-Beattie-Bresnahan (BBB) score (Basso et al., 1995). The score system assigns a score of 21 to normal rats and 0 to completely paralyzed rats. Two experienced experimenters conducted evaluations at a consistent time in the morning on each test day. They independently assessed the BBB scores within 5 min to determine the hind limbs’ motor function recovery following SCI. In addition, the limb muscle strength in rats was evaluated using the inclined plane test (Rivlin and Tator, 1978). The test involved gradually increasing the angle between the horizontal plane and the inclined plane until the rats were unable to maintain a constant posture for more than 5 s. The maximum angle achieved before losing posture was recorded as the result of the inclined angle for the rats.

### 2.4 Quantitative real-time polymerase chain reaction (QPCR)

TRIZOL reagent (No. A33250, Invitrogen) was used to extract RNA and reverse transcribed into cDNA according to the manufacturer’s instructions. The total RNA extracted is approximately 1 μg, with a reverse transcription system of 20 μl. The cDNA product after reverse transcription is diluted five times before PCR amplification. The QPCR was performed using SYBR green dye (No. #RR067A, Takara) in a thermal cycler with the following parameters: an initial denaturation step at 95°C for 30 min; followed by 40 cycles at 95°C for 5 s and 60°C for 30 s. Each sample underwent the entire experimental process independently. The QPCR results were quantified by 2^–ΔΔCT^. The forward primer of GAPDH is 5′-ACA GCA ACA GGG TGG TGG AC-3′; the reverse primer of GAPDH is 5′-TTT GAG GGT GCA GCG AAC TT-3′. The forward primer of GAL3 is 5′-AGG CTC CTC CTA GTG CCT AT-3′; the reverse primer of GAL3 is 5′-CCT CCA GGC AAG GGC ATA TC-3′. The forward primer of CDC42 is 5′-AAA GAA AAG TGG GTG CCT GAG-3′; the reverse primer of CDC42 is 5′-AGC AGT CTC TGG AGT GAT AGG -3′.

### 2.5 Co-immunoprecipitation (Co-IP) and western blot

The protein samples were obtained from rat spinal cord tissue and neurons, and the total soluble protein concentration was determined. The protein content of each experimental sample was approximately 100 μg. An immunoprecipitation experiment was conducted using the Co-IP kit (No.88804, Invitrogen). The whole cell lysate was pre-absorbed with protein A + G agarose beads (No.78610, Invitrogen) for 1 h, followed by high-speed centrifugation to remove any non-specifically bound proteins on protein A + G. Subsequently, the samples were incubated overnight at 4°C with the designated antibodies with gentle rotation. Protein samples were separated on 10% Tris- sodium dodecyl glycinate-polyacrylamide gel and transferred onto an immobile-PSQ transfer membrane (Merck Millipore, Darmstadt, Germany). Different membranes were mixed with GAPDH (1:1000, mouse IgG; No.10494, Proteintech), GAL3 (1:10000, rabbit IgG; No. 60207, Proteintech), CDC42 (1:1000, rabbit IgG; No.10155, Proteintech), ATG7 (1:1000, rabbit IgG; No.10088, Proteintech), P62 (1:1000, rabbit IgG; No.18420, Proteintech), and LC3 (1:1000, rabbit IgG; No. ab51520, Abcam). Then, the membrane was incubated with goat secondary antibody coupled with horseradish peroxidase against mouse IgG (1:10000; No. SA00001-1, Proteintech) or rabbit IgG (1:10000; No. SA00001-2, Proteintech). The signal was developed using a hypersensitive ECL chemiluminescence kit (No. G2020, Servicebio). Density measurement is performed by ImageJ software (version 1.53t, NIH, United States) to quantify the signal intensity.

### 2.6 Immunofluorescence

Rats were euthanized by cardiac puncture under terminal CO_2_ anesthesia (65% vol/min) with avertin (250 mg/kg), perfused with 0.9% saline, followed by 4% paraformaldehyde (pH 7.2). Then, the injured segment of the spinal cord was immediately dissected and separated, then fixed in 4% paraformaldehyde for 24 h before embedding into the paraffin section. After dewaxing the paraffin sections and performing heat antigen retrieval, immunofluorescence staining was performed. Next, the steps of immunofluorescence staining of paraffin sections or cell slides of tissues were the same. After incubation with blocking solution, samples were co-incubated overnight at 4°C with two primary antibodies simultaneously (a target protein antibody and a cellular marker antibody). All subsequent experimental procedures were performed under light-protected conditions: the tissues were washed with PBST three times, followed by incubation with corresponding secondary antibodies. Lastly, staining with DAPI (4’,6-diamidino-2-phenylindole) was performed for imaging. The primary antibody used targeted the following proteins: NeuN (1:500, mouse IgG; No.26975, Proteintech), GAL3 (1:200, mouse IgG; No. 60207, Proteintech), and CDC42 (1:200, rabbit IgG; No.10155, Proteintech). The secondary antibodies used were: Alexa fluor 488-implicated Donkey Anti-Rabbit IgG (1: 1000; No. SA00013-1, Proteintech) and Cy3–conjugated Affinipure Goat Anti-Rabbit IgG (1:1000; No. SA00009-2, Proteintech). Immunofluorescence images were captured using a fluorescence microscope (Carl Zeiss, Jena, Germany). Immunofluorescence intensity was quantified using ImageJ by calculating fluorescence intensity coefficients (FIC) for each group: (background-subtracted target protein intensity)/(background-subtracted cellular marker intensity). Statistical comparisons were made between experimental and control group FIC ratios, with cellular marker normalization eliminating positive cell number effects.

### 2.7 Extraction and culture of spinal cord neurons

One-day-old Sprague-Dawley rats were purchased from the Experimental Animal Center of Nantong University, and spinal cord neurons were isolated following these procedures: (1) Neonatal rats were euthanized with CO_2_ and immediately disinfected with 75% alcohol followed by decapitation. The spinal cord adventitia was carefully removed using a stereoscopic microscope in pre-cooled L-15 buffer (No. 11415064, Invitrogen). Subsequently, L-15 was aspirated, and the spinal cord was cut into pieces using 0.125% trypsin. The cord fragments were digested at 37°C for 30 min with gentle agitation every 10 min. (2) After tryptase treatment, the solution was removed, and the digestion was halted by adding Duchenne’s modified Eagle medium (DMEM, No. 11320033, Invitrogen) with 10% Fetal Bovine Serum (No. 30067334, Invitrogen) and 1% Penicillin-Streptomycin (No. C0222, Beyotime). After centrifugation at 1,000 rpm for 3 min, L-15 was added again to gently wash the resulting precipitate, followed by another centrifugation at 1,000 rpm for 3 min. (3) Gentle mixing of L-15 was followed by filtration of all liquids using a 70 μm sieve (No.FSTR070, Beyotime). The filtered liquids were carefully added in a 1:1 ratio on the upper layer of a motor neuron separation solution consisting of Nycoprep 1.077 (15%) (No.1114551, Axis-Shield), Hibernate-E medium (83%) (No. A1247601 Invitrogen), and Brewer’s B27 (2%) (No. 05711, Stemcell) (immiscible). Subsequently, the mixture was centrifuged at 2,000 rpm for 15 min. (4) The middle layer at the liquid surface was extracted and transferred into a new centrifuge tube. After centrifugation at 1,000 rpm for 3 min, the supernatant was discarded. Neurobasal-A culture medium (No. 10888022, Invitrogen), containing 2 mmol/L L-glutamine and 2% concentration of B27 neuron-specific additive, with final concentrations of 100 U/mL and 0.1 mg/mL for penicillin and streptomycin, respectively, were added. The solution was then inoculated into a culture plate for cell culture at 37°C in an incubator containing 5% carbon dioxide. (5) Replacement of the medium occurred 24 h post-plating and continued every 2 days. To enhance the neuron purity, Ara-C (No.HY-13605, MedChemExpress) at a concentration of 0.05 mg/mL was added 24 h after plating. (6) For the nerve injury model, after 3 days of cell culture, the experimental group and the cell transfection control group (the siR-Con group) were stimulated with a culture solution containing 10 μM Glutamate (No. 56-86-0, Sigma-Aldrich) for 1 h (Shahsavani et al., 2021; Yan et al., 2024), while the control group received an equal volume of phosphate buffer saline. Subsequently, both groups were cultured for an additional 16 h in a Neurobasal-A culture medium for glutamate-free neurons.

### 2.8 High-throughput sequencing of spinal cord neurons

Total RNA was extracted from neonatal Sprague-Dawley rat spinal cord neurons using TRIZOL reagent (Invitrogen) in collaboration with Allwegene Co. Ltd (Nanjing, China). Sequencing was carried out using the Illumina high-throughput sequencing platform (HiSeqTM2500/4000). A total of six samples were sequenced, including three samples from neurons as the transfection control group and three samples from neurons with GAL3 knocked down. The data provided by the company underwent batch effect removal, standardization, and correction for intra-group differences, resulting in an expression matrix. The detailed sequencing steps conducted by the sequencing company were provided in the “Supplementary Figure 1.”

### 2.9 Bioinformatics analysis

We downloaded the datasets of GSE2599 (Aimone et al., 2004), GSE20907 (Siebert et al., 2010), GSE45006 (Chamankhah et al., 2013), and GSE174549 (Li E. et al., 2022) from the Gene Expression Omnibus Datasets (GEO) available at https://www.ncbi.nlm.nih.gov/geo/. Table 2 displays the basic information for these four datasets. The 60 samples were re-divided into two groups based on the relative expression level of GAL3, and the role of GAL3 in SCI was investigated by single-gene analysis. Before dataset analysis, batch effects were removed, and standardization pretreatment was conducted. The R software (v4.3.0^1^) was used to analyze and visualize data. The r package used to remove the batch effect is sva (Ge et al., 2011) (v3.46.0^2^). After obtaining the expression matrix, limma package (Ritchie et al., 2015) (v3.54.2^3^) was used for differential expression analysis. The screening criteria of differential expression genes (DEGs) are |logFC| > 1 and *P*-value < 0.05. We used Gene Set Enrichment Analysis (GSEA) (Han et al., 2019) as a calculation method to determine whether a set of *a priori*-defined genes show statistically significant consistent differences between two biological states by clusterProfiler (Wu et al., 2021) (v4.0.5^4^). We used ggplot2 (Ito and Murphy, 2013) (v3.4.1^5^) to screen out the candidate genes and used the STRING^6^ website and the Cytoscape software (v3.10.0^7^) to analyze the candidate genes by Protein-Protein Interaction Networks (PPI)^47^.

**TABLE 2:** Basic information of four datasets.

GSE ID	Platform	Sham vs. SCI	Year	Gender	Weight/ age	Species	Injury site	Damage details
GSE2599	GPL85	3-3	2004	Female	165–200 g	*Rattus norvegicus*	Thoracic 8	The T7 and T9 spinal processes were clamped in a spinal frame, and contusions were made by rapidly displacing the cord 1.0 mm (moderate injury).
GSE20907	GPL6247	4-20	2010	Female	77 ± 10 days	*Rattus norvegicus*	Thoracic 9	The animal was positioned and secured into the frame of an NYU Impactor by clamping the T8 and T11 spinous processes, followed by a moderate spinal contusion injury, dropping a 10 g rod from a height of 25 mm using the MASCIS protocol.
GSE45006	GPL1355	4-20	2013	Female	250 g	*Rattus norvegicus*	Thoracic 7	Rats underwent a T6–T8 laminectomy and then received a 35 g clip (Walsh) moderate to severe compression injury at T7 for 1 min.
GSE174549	GPL25947	3-3	2022	Female	250 g	*Rattus norvegicus*	Thoracic 8–10	An impactor weighing 10 g was dropped vertically from a height of 50 mm onto the exposed T9 spinal cord surface, causing a contusion injury.

### 2.10 Collection of human serum

Blood samples were collected from 13 patients diagnosed with SCI, and admitted between January 2022 and December 2023, on the first day of admission. Serum was extracted from these samples after high-speed centrifugation and stored at −80°C. The screening criteria for patients’ inclusion in the SCI group were as follows: diagnosis of spinal cord injury confirmed through CT, MRI, and other imaging examinations, in according with the International Standard for Neurological Classification of SCI (revised in 2019) (ASIA and ISCoS International Standards Committee, 2019); Absence of spinal malignant tumor, and tuberculosis-related pathological SCI, or visceral injury; No concurrent diseases such as craniocerebral injury. Additionally, 13 serum samples from individuals without SCI were collected as the control group, and the screening criteria included: Age difference from the SCI patients within 5 years and absence of underlying health conditions. All subjects volunteered for inclusion in this study and provided informed consent. Approval for this study was obtained from the Medical Ethics Committee of Nantong First People’s Hospital (Protocol number: 2020KY036).

### 2.11 Enzyme-linked immunosorbent assay (ELISA)

After removing the cells from the cell culture incubator, collect the supernatant. Then gently wash the spinal cord neurons with PBS at 4°C. Add an appropriate amount of cell lysis solution mixed with 1/100 protease inhibitor and 1/50 phosphatase inhibitor, gently scrape off the cells, and place them on ice for 15 min for lysis. Centrifuge at 4°C and 13,000 rpm for 20 min. Collect the protein supernatant. The ELISA kit (GAL3, No. E0497h, ElAab; and CDC42, No. E0321r, ElAab) were utilized to measure the expression levels of GAL3 and CDC42 in human serum, rat serum, primary neuron cell lysate, and primary neuron cell supernatant. The samples were stored at −80°C before measurement. The optical density at 450 nm was calculated by subtracting the background value, and the standard curve was conducted.

### 2.12 Data analysis

All data were analyzed using GraphPad Prism 9.0 (GraphPad Software Inc., California, United States). All data are presented as mean ± standard error and were derived from at least three independent repeated experiments. The difference in BBB locomotor scores and inclined plane degrees between groups were assessed using two-way Repeated Measurement ANOVA. One-way ANOVA was used to evaluate the significance of the differences between groups for other data. The HSD (Honestly Significant Difference) test was used as a post hoc test following ANOVA. Unpaired Student’s *t*-test was used for comparisons between the two groups. A significant level of *P* < 0.05 was considered statistically significant.

## 3 Results

### 3.1 SCI increases GAL3 expression in spinal neurons

**FIGURE 1:**
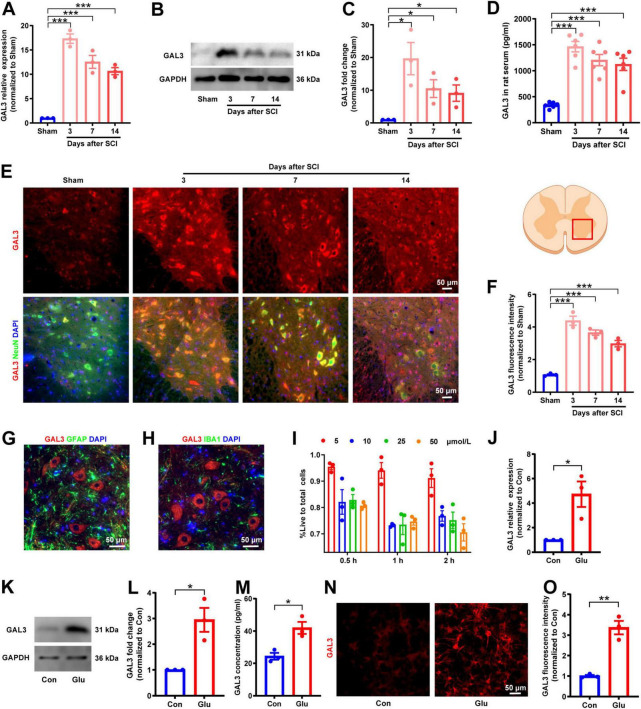
Spinal cord injury (SCI) increases galectin-3 (GAL3) expression in spinal neurons. **(A)** Relative GAL3 mRNA expression in the spinal cord after SCI. One-way ANOVA, *n* = 3/group. **(B)** Western blot analysis of GAL3 protein after SCI. **(C)** Statistical data show relative GAL3 protein expression after SCI. One-way ANOVA, *n* = 3/group. **(D)** Enzyme-linked immunosorbent assay (ELISA) detection of GAL3 protein levels in rat serum after SCI. One-way ANOVA, *n* = 6/group. **(E)** Immunofluorescence microscopy reveals GAL3 co-localization with NeuN post-SCI. **(F)** Fluorescence intensity of GAL3 after SCI. One-way ANOVA, *n* = 3/group. **(G,H)** Immunofluorescence double staining of GAL3 and GFAP **(G)** or IBA1 **(H)** after SCI. **(I)** Determination of optimal glutamate concentration and duration using CCK8 assay. **(J)** Relative GAL3 mRNA expression in the glutamate-stimulated spinal cord neurons. Unpaired Student’s *t*-test, *n* = 3/group. **(K)** Western blot analysis of GAL3 protein in neuronal injury model. **(L)** Relative GAL3 protein expression in neuronal injury model. Unpaired Student’s *t*-test, *n* = 3/group. **(M)** ELISA detection of GAL3 in cell supernatant of neuronal injury model. Unpaired Student’s *t*-test, *n* = 3/group. **(N)** Immunofluorescence microscopy showing GAL3 expression in neuronal injury model. **(O)** Quantification of GAL3 fluorescence intensity in neuronal injury model. Unpaired Student’s *t*-test, *n* = 3/group. *P < 0.05, **P < 0.01, ***P < 0.001.

After successfully establishing the rat SCI model, we examined GAL3 expression in spinal cord tissue and serum. The mRNA and protein were extracted at intervals of 3rd, 7th, and 14th days post-SCI. QPCR and Western Blot showed that the mRNA level and protein expression level of GAL3 were increased after SCI and peaked on day 3 after injury, gradually decreasing thereafter (Figures 1A–C). Notably, GAL3 level in serum was also increased post-SCI (Figure 1D). Immunofluorescence analysis revealed that GAL3 was co-localized with neurons in the ventral horn of the spinal cord (Figure 1E), notably significant at 3rd, 7th, and 14th days post-SCI (Figure 1F). However, GAL3 did not co-localize with astrocyte marker GFAP (glial fibrillary acidic protein) (Figure 1G) or microglia marker IBA1 (Figure 1H) post-SCI, suggesting a potential involvement of neuronal GAL3 in SCI pathogenesis.

Given that extracellular glutamate triggers excitotoxic neuronal cell death (Tehse and Taghibiglou, 2019), and is a significant factor in SCI-associated neurological dysfunction (Hausmann, 2003; Ribeiro and de Wit, 2017), we used glutamate to induce a neuron injury model (Rong et al., 2019; Shahsavani et al., 2021). Cell Counting Kit-8 (CCK8) experiments showed that 10 μM glutamate for 1 h yield the most suitable injury models (Figure 1I). QPCR showed that the mRNA of GAL3 was higher than that of the control group (Figure 1J). Western blot showed upregulated GAL3 protein levels (Figures 1K, L). ELISA further supported increased GAL3 secretion (Figure 1M). The immunofluorescence revealed heightened GAL3 fluorescence intensity in neurons (Figures 1N, O). These results collectively indicate GAL3 involvement in spinal cord neuron injury mechanisms after SCI.

### 3.2 GAL3 contributes to SCI-induced motor impairment

**FIGURE 2:**
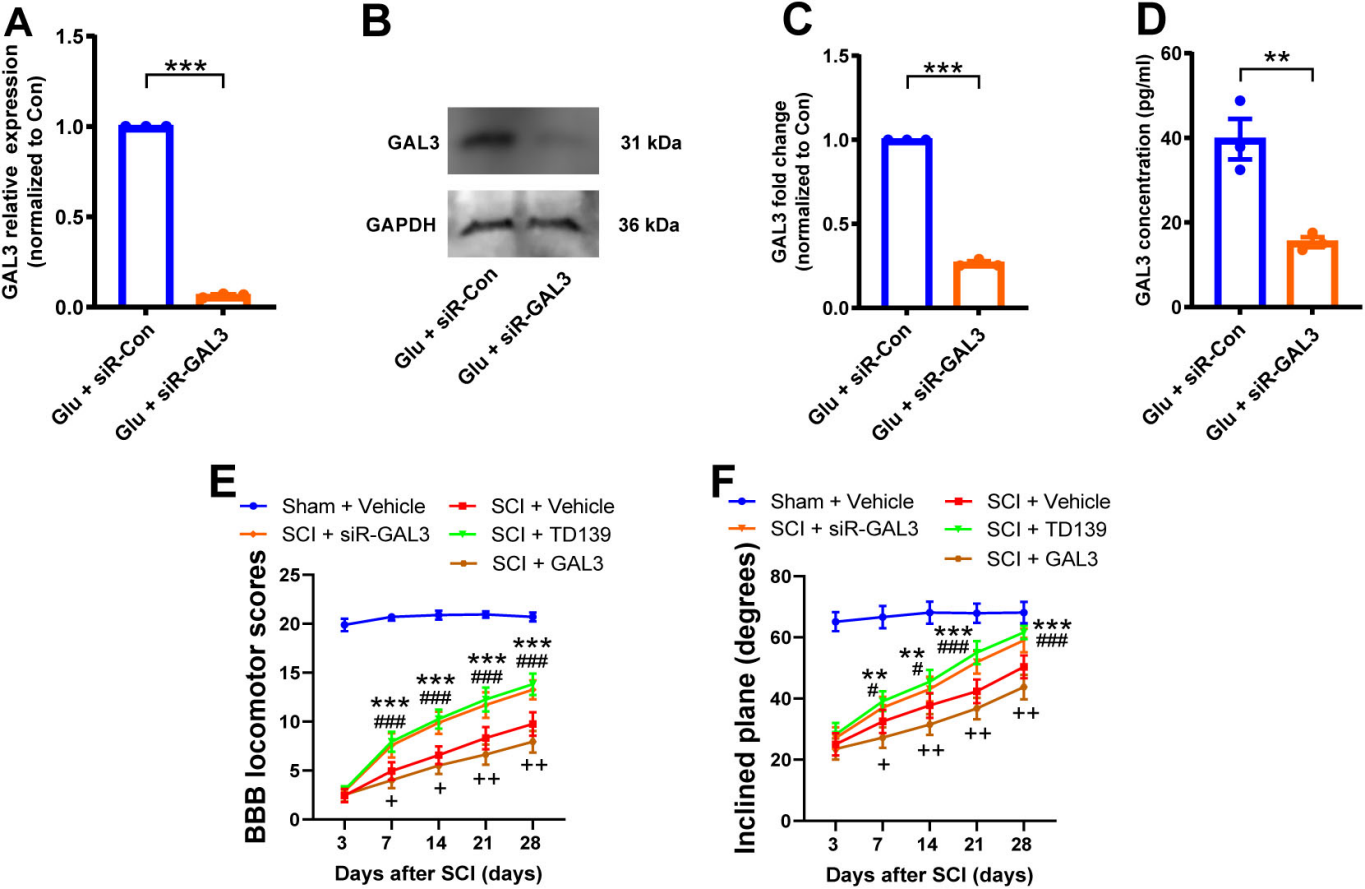
Galectin-3 (GAL3) contributes to spinal cord injury (SCI)-induced motor impairment. **(A)** The mRNA level of GAL3 after siR-GAL3 treatment. Unpaired Student’s *t*-test, *n* = 3/group. **(B)** Western blot shows the protein level of GAL3 after siR-GAL3 treatment. **(C)** Statistical data show the knockdown of GAL3 by siRNA. Unpaired Student’s *t*-test, *n* = 3/group. **(D)** Enzyme-linked immunosorbent assay (ELISA) shows the secretory GAL3 in the supernatant of neurons after siRNA treatment. Unpaired Student’s *t*-test, *n* = 3/group. **(E)** The Basso-Beattie-Bresnahan (BBB) locomotor scores were increased after siR-GAL3 or inhibitor treatment. Two-way Repeated Measures ANOVA, *n* = 8/group. **(F)** The inclined plane angles were increased after siR-GAL3 or inhibitor treatment. Two-way Repeated Measures ANOVA, *n* = 8/group. When SCI + siR-GAL3 group was compared with SCI + Vehicle group, **P < 0.01, ***P < 0.001; when SCI + TD139 group was compared with SCI + Vehicle group, #*P* < 0.05, ###*P* < 0.001; when SCI + GAL3 group was compared with SCI + Vehicle group, +*P* < 0.05,++*P* < 0.01.

To explore the specific role of GAL3 in the mechanism of spinal cord neuron injury, we used siRNA to knock down the GAL3 gene in spinal cord neurons under glutamate stimulation. The knock-down efficiency of siR-GAL3 resulted in a 90% reduction in GAL3 mRNA levels (Figure 2A), a 75% decrease in GAL3 protein expression (Figures 2B, C), and a 60% decline in secreted GAL3 (Figure 2D).

Subsequently, we administrated the siR-GAL3 or GAL3 inhibitor, TD139, into the injured spinal cord of rats for 7 consecutive days to observe motor function recovery post-SCI. Starting from the 7th-day post-SCI, both experimental groups displayed increased BBB scores compared to the simple SCI group (Figure 2E). Similarly, the degrees of the inclined plane angles also exhibited notable improvement (Figure 2F). These data suggest that knockdown or inhibition of GAL3 effectively promotes the recovery of motor function in rats following SCI. Interestingly, when administering GAL3 recombinant protein continuously for 7 days in the injured spinal cord of rats, we observed that starting from the 7th day following SCI, compared to the SCI + Vehicle group, the rats in the SCI + GAL3 group showed a decrease in BBB scores and incline angle, indicating that the injection of GAL3 hindered the recovery of motor function in SCI.

### 3.3 GAL3 regulates neuronal autophagy

**FIGURE 3:**
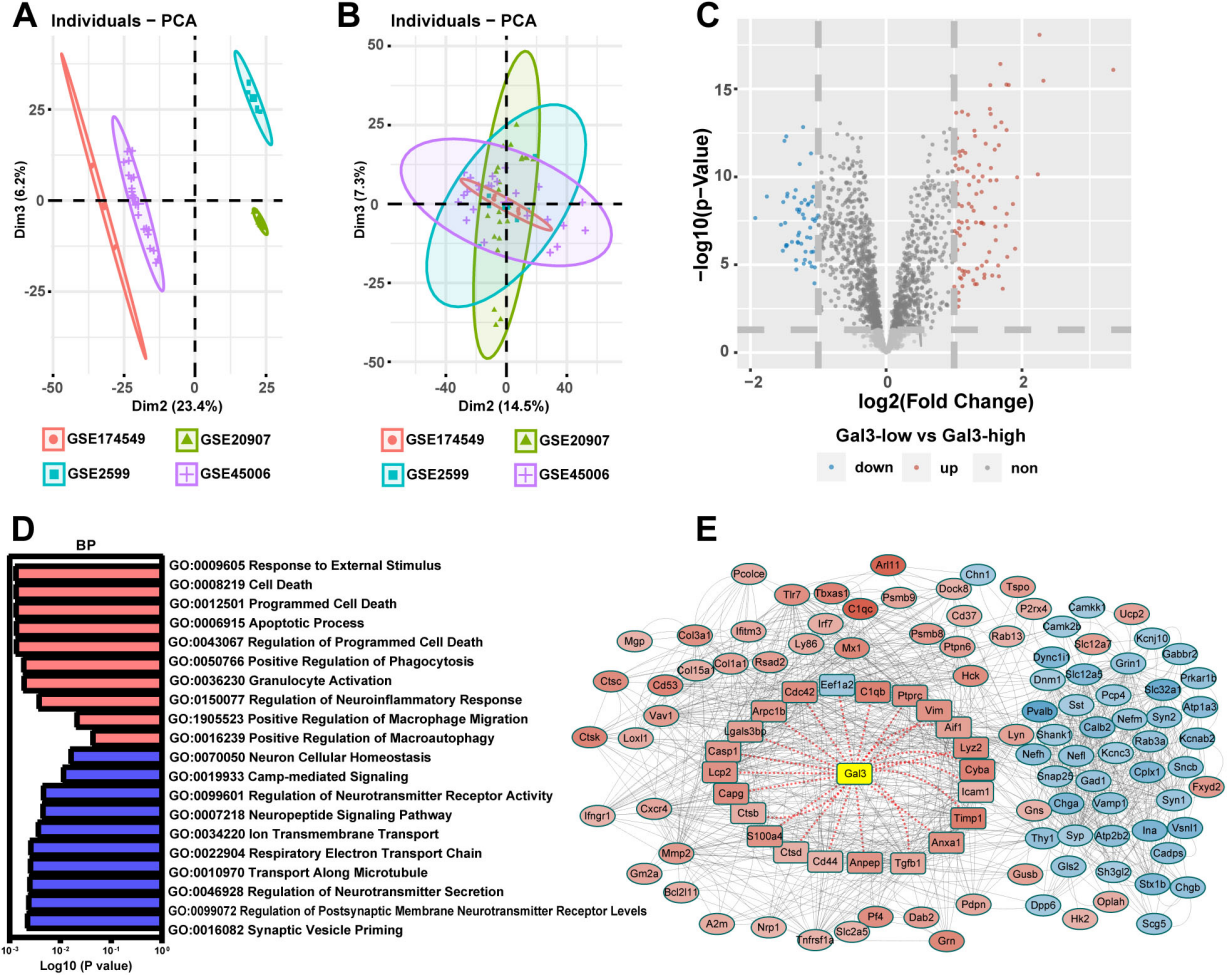
Galectin-3 (GAL3) is closely related to programmed cell death after spinal cord injury (SCI). **(A)** The four datasets before the batch effect were removed. **(B)** The four datasets after the batch effect were removed. **(C)** Volcano map shows DEGs in the SCI dataset. **(D)** Biological Process (BP) analysis of Gene Set Enrichment Analysis (GSEA) in the SCI dataset. Each column represents the *P*-value score of the pathway between the Sham group and the SCI group, with red indicating upregulation of the pathway in the SCI group, and blue indicating downregulation. **(E)** Protein-Protein Interaction Networks (PPI) analysis of differentially expressed genes (DEGs) in SCI dataset. In the PPI nodes, red signifies an increase in expression level, while blue indicates a decrease. The intensity of the color corresponds to the magnitude of the differential expression, with darker shades representing a higher differential expression multiple.

To unravel the role of GAL3 in the mechanism of SCI, we conducted a single-gene bioinformatics analysis of GAL3 involvement by rat SCI datasets obtained from GEO. Before formal dataset analysis, the batch effect was removed (Figures 3A, B). After batch effect removal and standardization, we obtained expression levels for 2,240 genes. Subsequent differential expression analysis revealed 158 differentially expressed genes (DEGs), of which 101 were up-regulated and 57 were down-regulated (Figure 3C). Biological Process (BP) analysis via GSEA, demonstrated significant enrichment of the highly expressed gene set functioned in pathways associated with programmed cell death mechanisms, such as *GO:0008219 Cell Death*, *GO:0012501 Programmed Cell Death*, *GO:0006915 Apoptotic Process* and *Go: 0016239 Positive Regulation of Macroautophagy* (Figure 3D).

To determine the relationship between these 158 DEGs and GAL3, we conducted the protein-protein interaction (PPI) analysis using the STRING website (Figure 3E). This analysis revealed evidence of interaction between GAL3 and 22 core nodes, with 21 of the core nodes showing up-regulated expression, except for the down-regulation of Eef1a2. These results strongly indicate that GAL3 is closely related to programmed cell death after SCI.

**FIGURE 4:**
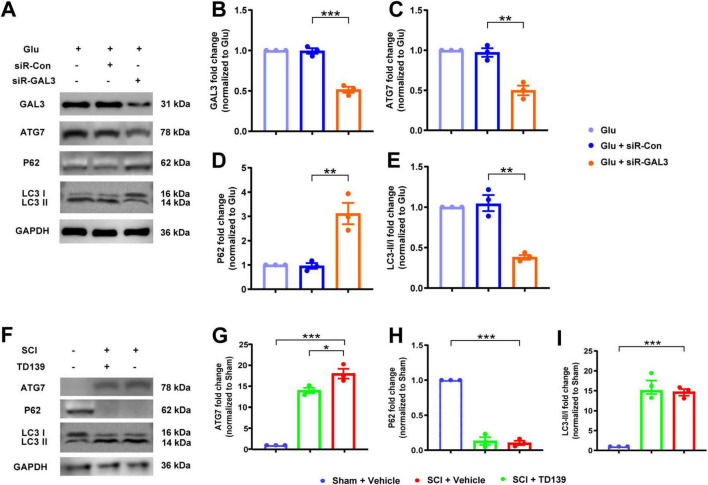
Galectin-3 (GAL3) regulates neuronal autophagy. **(A)** Western blot analysis of GAL3 and neuronal autophagy markers ATG7, P62, and LC3 II/I in neurons. **(B-E)** Quantification of western blot detection of GAL3 **(B)**, ATG7 **(C)**, P62 **(D)**, and LC3 II/I **(E)** in neurons. One-way ANOVA, *n* = 3/group. **(F)** Western blot analysis of GAL3 and neuronal autophagy markers ATG7, P62, and LC3 II/I in the spinal cord of rats. **(G–I)** Quantification of western blot detection of ATG7 **(G)**, P62 **(H)**, and LC3 II/I **(I)** in the spinal cord of rats. One-way ANOVA, *n* = 3/group. *P < 0.05, **P < 0.01, ***P < 0.001.

Considering the pivotal role of autophagy in programmed cell death, we validated whether GAL3 functions through autophagy after SCI. Initially, we used glutamate to stimulate neurons treated by siR-GAL3 (Figure 4A). In comparison with the siR-Con group, the siR-GAL3 group displayed significantly reduced GAL3 expression (Figure 4B), decreased ATG7 expression (Figure 4C), increased P62 expression (Figure 4D), and a decreased LC3 II/I ratio (Figure 4E).

We then examined autophagy in the spinal cord after injection with TD139. We extracted tissue protein and serum from rats in the Sham group, the SCI group, and the SCI + TD139 group on the 7th day after SCI (Figure 4F). Compared with the Sham group, expression levels of ATG7 in the SCI group increased (Figure 4G), whereas the P62 level decreased (Figure 4H), and the LC3 II/I ratio increased (Figure 4I), suggesting elevated autophagy level post-SCI. Furthermore, compared to the SCI group, expression levels of ATG7 decreased in the SCI + TD139 group (Figure 4G), while P62 expression and LC3 II/I ratio remained unchanged (Figures 4H, I). These results indicate that inhibition of GAL3 reduced autophagy levels at the injured site in rats.

### 3.4 GAL3 interacts with CDC42 to regulate neuronal autophagy

**FIGURE 5:**
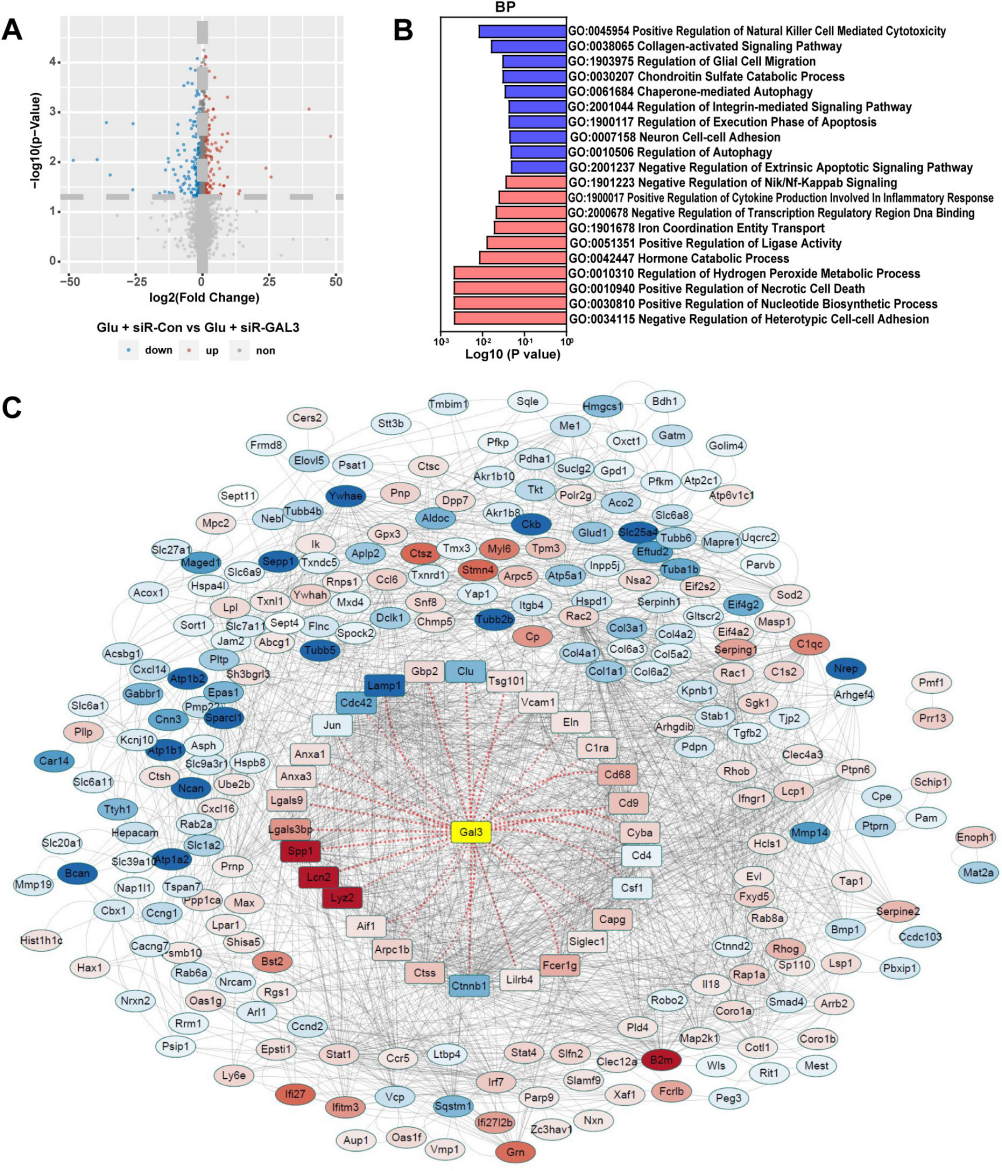
Sequencing analysis of spinal cord neurons with galectin-3 (GAL3) knocked down. **(A)** Volcano map shows differential expression genes (DEGs) in the neuron dataset. **(B)** Biological process (BP) analysis of Gene Set Enrichment Analysis (GSEA) in the neuron dataset. Each column represents the *P*-value score of the pathway between the Sham group and the spinal cord injury (SCI) group, with red indicating upregulation of the pathway in the SCI group, and blue indicating downregulation. **(C)** The Protein-Protein Interaction Networks (PPI) analysis of DEGs in the neuron dataset. In the PPI nodes, red indicates that the expression level increases and blue indicates that the expression level decreases. The darker the color, the greater the differential expression multiple.

**FIGURE 6:**
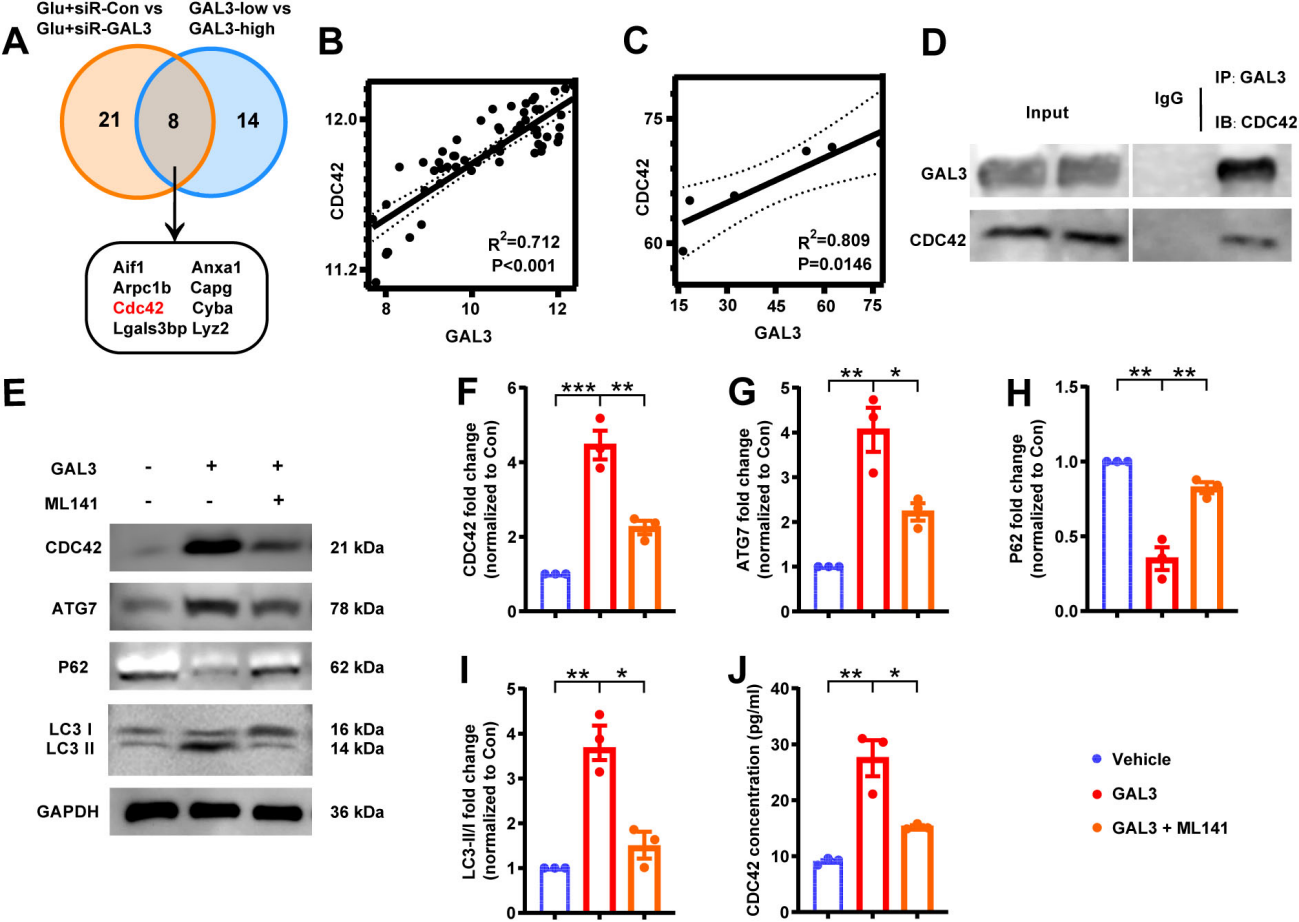
Galectin-3 (GAL3) interacts with Cell-division-cycle-42 (CDC42) to regulate neuronal autophagy. **(A)** Intersected 29 core nodes from the neuron dataset with 22 core nodes from the spinal cord injury (SCI) dataset by the Venn diagram. **(B)** Correlation analysis between GAL3 and CDC42 expression level in SCI dataset. **(C)** Correlation analysis between GAL3 and CDC42 expression level in the neuron dataset. **(D)** Co-immunoprecipitation (Co-IP) shows a direct interaction between GAL3 and CDC42 in the glutamate-induced neuronal damage model. **(E)** Western blot shows the expression of CDC42, ATG7, P62, and LC3 II/I. **(F–I)** Quantification of western blot detection of CDC42 **(F)**, ATG7 **(G)**, P62 **(H)**, and LC3 II/I **(I)**. One-way ANOVA, *n* = 3/group. **(J)** Enzyme-linked immunosorbent assay (ELISA) detection of CDC42 in cell supernatant of GAL3-injury model. Unpaired Student’s *t*-test, *n* = 3/group. *P < 0.05, **P < 0.01, ***P < 0.001.

To delve deeper into the specific role of GAL3 in the mechanism of spinal cord neuron injury, we extracted RNA from Glu + siR-Con and Glu + siR-GAL3 neurons for sequencing analysis. The volcano plot revealed 316 DEGs, with 151 up-regulated and 165 down-regulated genes (Figure 5A). Further, BP of GSEA showed significant enrichment in genes linked to autophagy mechanisms like *Go: 0010506 Regulation of autophagy* and *Go: 0061684 Chaperone-mediated autophagy* (Figure 5B). To establish connections between these 316 DEGs and GAL3, PPI analysis via the STRING website identified 29 core nodes interacting with GAL3, where 25 core nodes exhibited up-regulation, excluding Clu, Lamp1, Cdc42, and Ctnnb1 (Figure 5C). Subsequently, by overlapping 29 core nodes with 22 core nodes from the SCI dataset, eight core genes were screened out using a Venn diagram (Figure 6A). However, only CDC42 showed an upregulation with GAL3 in the SCI dataset, and down-regulated in the siR-GAL3-treated neuron dataset. Additionally, the expression level of GAL3 positively correlated with CDC42 expression in both the SCI dataset (R^2^ = 0.712, *P* < 0.001) (Figure 6B) and the siR-GAL3-treated neuron dataset (R^2^ = 0.809, *P* = 0.0146) (Figure 6C). Furthermore, Co-IP experiments confirmed the interaction between GAL3 and CDC42 (Figure 6D).

Then we stimulated neurons with GAL3 and detected the expression levels of CDC42 and autophagy markers. Western blot analysis showed, compared to the control group, expression levels of CDC42 and ATG7 increased, P62 expression decreased, and the LC3 II/I ratio increased in the GAL3 group (Figures 6E–I). However, the GAL3 + ML141 (the CDC42 molecular inhibitor) group displayed lower autophagy levels than the GAL3 group (Figures 6E–I). In addition, we detected the expression level of CDC42 in the supernatant of neurons, which followed a similar trend as the western blot (Figure 6J). These data indicate that GAL3 promotes neuron autophagy, and inhibition of CDC42 suppresses GAL3-induced autophagy.

### 3.5 SCI increases CDC42 expression in spinal neurons

**FIGURE 7:**
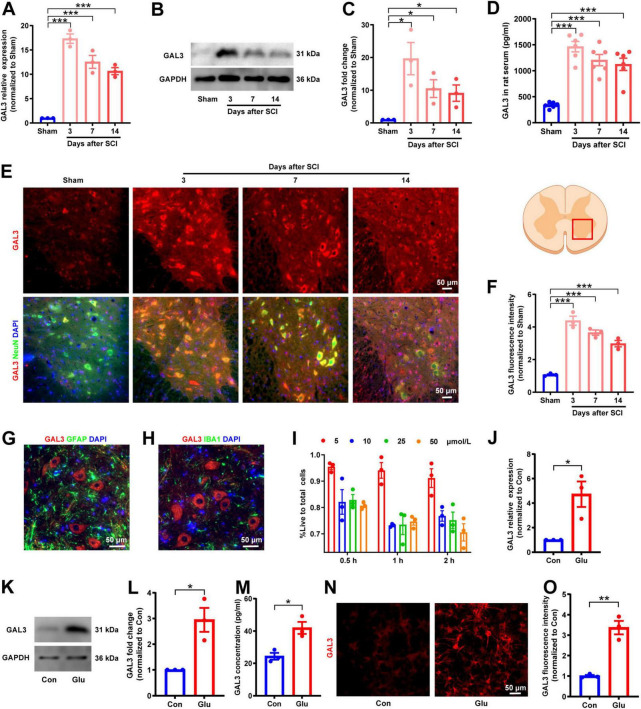
Spinal cord injury (SCI) increases Cell-division-cycle-42 (CDC42) expression in spinal neurons. **(A)** Relative expression level of CDC42 mRNA in rats after SCI. One-way ANOVA, *n* = 3/group. **(B)** Western blot analysis of CDC42 protein in rats after SCI. **(C)** Relative expression level of CDC42 protein in rats after SCI. One-way ANOVA, *n* = 3/group. **(D)** Co-localization of CDC42 and neuronal marker NeuN after SCI observed by immunofluorescence microscopy. **(E)** Fluorescence intensity of CDC42 after SCI in rats. One-way ANOVA, *n* = 3/group. **(F)** Double staining of CDC42 and astrocytes marker glial fibrillary acidic protein (GFAP) after SCI. **(G)** Double staining of CDC42 and microglial marker IBA1 after SCI. **(H)** Relative expression level of CDC42 mRNA in the glutamate-stimulated spinal cord neurons. Unpaired Student’s *t*-test, *n* = 3/group. **(I)** Western blot analysis of CDC42 protein in neurons. **(J)** Relative expression level of CDC42 protein in the neuron injury model. Unpaired Student’s *t*-test, *n* = 3/group. **(K)** Expression of CDC42 observed in the neuron injury model by immunofluorescence microscopy. **(L)** Fluorescence intensity of CDC42 in the neuron injury model. Unpaired Student’s *t*-test, *n* = 3/group. *P < 0.05, **P < 0.01, ***P < 0.001.

We detected the expression level of CDC42 in rats post-SCI. We found an increase in both the mRNA level and protein level of CDC42 in the spinal cord tissue after SCI, peaking on the third day post-SCI, followed by a decrease over time (Figures 7A–C). Immunofluorescence analysis of spinal cord tissue showed co-localization of CDC42 within neurons in the ventral horn, statistically significant on the 3rd, 7th, and 14th days post-SCI (Figures 7D, E). Moreover, post 3rd-day of SCI immunofluorescence analysis showed a lack of co-localization between CDC42 and GFAP or IBA1 (Figures 7F, G). Similarly, in the glutamate-induced neuron injury model, both mRNA and protein expression levels of CDC42 were increased (Figures 7H-J), with an increased fluorescence intensity observed (Figures 7K, L), indicating a potential role of CDC42 in SCI.

### 3.6 CDC42 contributes to SCI-induced motor function impairment

**FIGURE 8:**
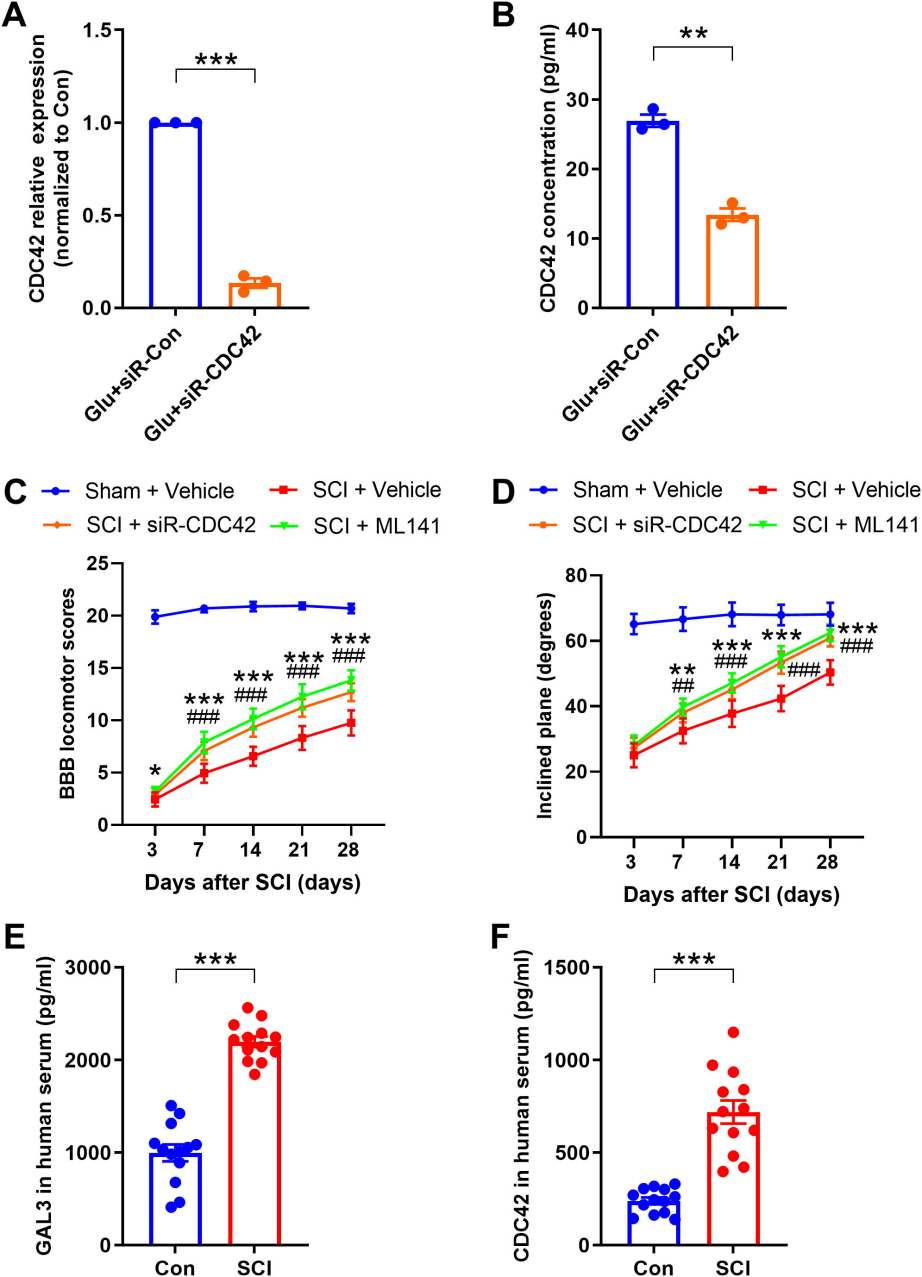
Cell-division-cycle-42 (CDC42) contributes to spinal cord injury (SCI)-induced motor function impairment. **(A)** The mRNA level after siR-CDC42 treatment. Unpaired Student’s *t*-test, *n* = 3/group. **(B)** Enzyme-linked immunosorbent assay (ELISA) shows the secretory CDC42 in the supernatant of neurons after siRNA treatment. Unpaired Student’s *t*-test, *n* = 3/group. **(C)** The Basso-Beattie-Bresnahan (BBB) locomotor scores were increased after siR-CDC42 and ML141 treatment. Two-way Repeated Measures ANOVA, *n* = 8/group. **(D)** The inclined plane angles were increased after siR-CDC42 and ML141 treatment. Two-way Repeated Measures ANOVA, *n* = 8/group. When SCI + siR-CDC42 group was compared with SCI + Vehicle group, *P < 0.05, **P < 0.01, ***P < 0.001; when SCI + ML141 group was compared with SCI + Vehicle group, ##*P* < 0.01, ###*P* < 0.001. **(E,F)** Detection of the protein expression level of galectin-3 (GAL3) **(E)** and CDC42 **(F)** in serum of healthy volunteers and SCI patients by ELISA. Unpaired Student’s *t*-test, *n* = 8/group. ***P < 0.001.

To explore the mechanism of CDC42 in SCI, we injected siR-CDC42 and the CDC42 molecular inhibitor ML141, into the injured site of SCI rats, observing the recovery of hind limb motor function. The knock-down efficiency of siR- CDC42 resulted in an 85% reduction in CDC42 mRNA levels (Figure 8A), and a 50% decline in secreted CDC42 (Figure 8B). Analysis of BBB locomotor scores and inclined plane experiments revealed that siR-CDC42 and ML141 injection indeed promoted the recovery of motor function in SCI rats (Figures 8 C, D).

Finally, to understand the expression trend of GAL3 and CDC42 in human serum post-SCI, we collected samples from 8 individuals with SCI and 8 healthy controls. We found higher levels of GAL3 and CDC42 in the serum of SCI patients compared to those in the control group (Figures 8E, F).

## 4 Discussion

In this study, we discovered an increase in GAL3 expression in spinal neurons following SCI. Furthermore, both the knockdown and inhibition of GAL3 not only facilitated motor function recovery after SCI but also resulted in a reduction in neuronal autophagy. Through the application of bioinformatics analysis and Co-IP, we identified a novel interaction between GAL3 and CDC42. The administration of CDC42 inhibitor ML141 effectively countered GAL3-mediated enhancement of neuronal autophagy, leading to a decrease in the expression of autophagy markers, and an improvement in motor function recovery in rats with SCI. Notably, we detected heightened levels of GAL3 and CDC42 in the serum of SCI patients. These findings indicate the involvement of GAL3 and CDC42 in neuronal autophagy after SCI, underscoring the potential therapeutic targets of GAL3/CDC42 for enhancing recovery from SCI.

Previous studies demonstrated that GAL3 is upregulated in various human conditions such as Alzheimer’s disease and stroke (Al-Dalahmah et al., 2020; Tan et al., 2021), as well as in rodent models including Alzheimer’s disease, multiple sclerosis, stroke, and hypoxia/ischemia (Akazawa et al., 2004; Boza-Serrano et al., 2019; Walther et al., 2000; Young et al., 2014). In the mice model, GAL3 inhibitors have demonstrated significant efficacy in reducing renal injury in various conditions (Wang F. et al., 2023). Studies indicate mice lacking GAL3 in immune effector cells exhibited milder symptoms of type 1 diabetes (Mensah-Brown et al., 2009). Moreover, inhibition of GAL3 improved the conditions associated with nervous system diseases. In rats, GAL3 inhibition is linked to alleviated neuropathic pain after peripheral nerve injury (Ma et al., 2016). GAL3 knockout mice exhibited decreased stroke size and improved functional outcomes after a stroke (Rahimian et al., 2018; Soares et al., 2021), while loss of GAL3 function can diminish symptoms of neurodegenerative diseases such as Alzheimer’s (Soares et al., 2021). In addition, GAL3 knockout mice subjected to hypoxia/ischemia displayed reduced loss of neuronal cell volume, as well as diminished regional damage of the hippocampus and striatum (Doverhag et al., 2010). Our research has shown that inhibiting GAL3 facilitates the recovery of motor function after SCI, aligning with the previously mentioned findings.

Our GSEA of biological functions revealed a significant connection between elevated GAL3 expression and programmed cell death in our single-gene bioinformatics analysis of tissue samples post-SCI. Despite previous studies demonstrating GAL3’s involvement in autophagy mechanisms (Alvarez-Valadez et al., 2021; Zheng et al., 2023), there remains a plethora of investigations revealing conflicting associations between GAL3 and autophagic flux (Li et al., 2020; Mansour et al., 2022; Weng et al., 2018). One study indicated that shR-GAL3-treated melanoma cells exhibit a high level of accumulated LC3-II compared to melanoma cells expressing GAL3, resulting in an increased autophagy flux rate (Bustos et al., 2018). Zhao et al. observed more pronounced autophagy enhancement in GAL3 knockdown rat renal tubular epithelial cells than in cells overexpressing GAL3 (Zhao et al., 2023). In addition, a recent study found that treating THP-1 cells with siR-GAL3 led to an increase in the number of autophagosomes, a decrease in the expression level of P62, an increase in the expression level of Beclin1, and an elevated ratio of LC3-II/I (Wang Z. et al., 2023).

In contrast, some reports indicate that inhibiting GAL3 results in autophagy impairment under diverse experimental conditions (Da Silva et al., 2020; Jia et al’s., 2020; Khan et al., 2021; Yun et al., 2020). For example, Khan et al. (2021) reported that siR-GAL3 down-regulates autophagic vesicle trafficking, and Yun et al. (2020) observed a significant decrease in the number of autophagic vesicles following GAL3 inhibition using electron microscopy. Jia et al’s. (2020) research on lysosomal membrane repair provided additional support by demonstrating a reduction in autophagy efficiency in the absence of GAL3. Da Silva et al. (2020) reported that inhibiting GAL3 expression in pancreatic cell lines resulted in decreased levels of LC3. The outcomes of these studies suggest that the involvement of GAL3 in the formation of autophagic vesicle membranes and lysosomal membranes may contribute to the observed effects (Alvarez-Valadez et al., 2021). Our study aligns with these studies, as we observed that reducing GAL3 protein expression levels resulted in decreased autophagy levels in both *in vivo* and *in vitro* spinal neurons.

Autophagy is crucial in maintaining the balance between the production and degradation of cellular components under normal conditions. However, following nerve injury, autophagy dysregulation occurs, exhibiting both beneficial and detrimental roles due to dynamic environmental changes and underlying mechanisms (Wu and Lipinski, 2019). In this study, we used three autophagy markers-ATG7, P62, and LC3 to monitor the regulation of neuronal autophagy. ATG7, an indispensable enzyme in the autophagy process, collaborates with other ATG proteins to oversee the autophagic process (Collier et al., 2021). P62 levels serve as an indicator of autophagy-dependent degradation rate: low P62 levels indicate high autophagy flux (high degradation), while elevated P62 levels indicate a diminished autophagy flux (inhibition of degradation) (Katsuragi et al., 2015). Notably, the autophagy marker LC3-II exhibited an increase several hours post-SCI (Hao et al., 2013; Liu et al., 2015; Liu et al., 2018), persisting for about 60 days after the injury (Berliocchi et al., 2015; Munoz-Galdeano et al., 2018; Salminen et al., 2013; Zhang et al., 2013, 2014). Moreover, in rat and mouse SCI models, LC3 and P62 accumulate more prominently in motor neurons near the impact site compared to dorsal horn sensory neurons (Liu et al., 2015, 2018; Munoz-Galdeano et al., 2018).

By integrating the results into the PPI analysis from the SCI dataset and the GAL3-knocked-down neuron dataset, we identified a potential interaction between GAL3 and CDC42 following neuron injury. Furthermore, our correlation analyses demonstrated that GAL3 up-regulated CDC42 in the SCI dataset and down-regulated it in the GAL3-knocked-down neuron dataset. Therefore, we postulated that GAL3 might regulate CDC42 to play a role in neuronal autophagy. Previous studies indicated the association between members of the galectin family and Rho GTPases. GAL3 knockdown effectively inhibited RhoA expression in hypoxic non-small cell lung cancer cells (Kataoka et al., 2019) and GAL1 knockdown inhibited Ras expression in specific cell types (Shih et al., 2019). Additionally, GAL3 was found to promote RhoA expression in human umbilical vascular endothelial cells (Chen et al., 2019).

Imbalances in Rho GTPases are linked to synaptic irregularity in various nervous system diseases, including Alzheimer’s, Huntington’s, Parkinson’s, amyotrophic lateral sclerosis, and schizophrenia (Aguilar et al., 2017; Datta et al., 2015; Stankiewicz and Linseman, 2014). Inhibition of Rho GTPases has been proven to reduce secondary injury after SCI, enhance axonal regeneration, and promote neurological function recovery (Forgione and Fehlings, 2014; Roy et al., 2021). In addition, the activation of the RhoA-ROCK signaling pathway in glial and immune cells contributes to CNS neurodegeneration (Koch et al., 2018). CDC42, a member of Rho GTPases, triggers downstream signals through interaction with the primary regulator Ras, influencing inflammation (Mosaddeghzadeh and Ahmadian, 2021). The deficiency of CDC42 has been associated with improved allergic airway inflammation (Yang et al., 2019), and CDC42 inhibitors have demonstrated the reversal of pro-inflammatory effects in macrophages (Ma et al., 2022). In our study, we observed that CDC42 molecular inhibitor ML141 inhibited neuronal autophagy stimulated by GAL3. Importantly, our Co-IP experiments revealed an interaction between GAL3 and CDC42 *in vitro* after neuronal injury, suggesting that the identified interaction might represent a key pathway through which GAL3 regulates CDC42’s involvement in neuronal autophagy.

However, the mechanism of CDC42 in autophagy has been relatively understudied, and there is limited literature on this topic. CDC42 often assumes an important role in apoptosis as a downstream effector. It has been reported that silencing CDC42 significantly promoted apoptosis and inhibited proliferation in bladder cancer cell (Li G. et al., 2022). Knocking down the expression of CDC42 in planarian increased epidermal cell apoptosis without affecting cell division (Yujia et al., 2019). In addition, some *in vitro* studies have highlighted a significant increase in apoptosis levels in CDC42 knock-down renal podocyte cultures (Huang et al., 2016). Notably, an *in vivo* study indicated that the CDC42 inhibitor promoted tumor autophagy and apoptosis in a mouse rhabdomyosarcoma xenotransplantation model (Li et al., 2021). While these findings provide new insights into understanding the autophagy mechanism of neurons following SCI, further research is needed to unravel the intricate regulatory mechanisms between CDC42 and autophagy. Nonetheless, our data contribute to the growing body of evidence indicating a close association between CDC42 and neuronal autophagy.

Our study revealed an upregulation of CDC42 following neuronal injury in both *in vivo* and *in vitro*. Inhibition of CDC42 function with ML141 post-SCI not only promoted motor functional recovery but also led to a reduction in autophagy levels. Notably, strategies targeting Rho family-controlled pathways, including those focusing on CDC42, have garnered attention in cancer treatment (Murphy et al., 2021), infectious diseases (Hong et al., 2013), and neurodegeneration (Aguilar et al., 2017; Barcia et al., 2012; Rong et al., 2020). In a mouse model of Parkinson’s disease, a CDC42 inhibitor inhibited microglial reaction and protected neurons from phagocytosis (Barcia et al., 2012). Additionally, increased CDC42 and Rac1 were observed in specific neuron populations in the brain of AD patients (Zhu et al., 2000). Our research underscores the neuroprotective effects of GAL3 and CDC42 inhibitors post-SCI, highlighting their potential as therapeutic targets in the treatment of SCI.

The analysis of serum samples from SCI patients in our study revealed an upregulation of GAL3 and CDC42 expression. This discovery suggests their potential significance in both diagnostic and therapeutic applications for SCI management. Notably, ongoing clinical or preclinical research by pharmaceutical and biotechnology companies is exploring inhibitors and antagonists targeting GAL3, with some of these strategies showing promise (Ahmed et al., 2023).

This study has several limitations that warrant consideration. While we observed an inverse correlation between post-SCI autophagy levels and neurological functional recovery, establishing direct causality remains unproven. Despite the confirmed protective effect of autophagy in the experimental model of traumatic SCI (Saraswat Ohri et al., 2018; Sekiguchi et al., 2012; Tang et al., 2014), the nuances of post-SCI autophagy still require further molecular mechanism exploration. Autophagy after SCI is affected by various factors, including injury severity, timing, inflammatory responses, and treatment methods. A comprehensive understanding of how these factors impact autophagy can contribute to the development of more effective SCI rehabilitation strategies. Interestingly, although the existence of sex-dependent differences in post-SCI recovery remains controversial (Osimanjiang et al., 2022), our current understanding indicates no significant sex-based variation in locomotor functional recovery in rodent SCI models (McFarlane et al., 2020). However, this observation does not preclude the potential influence of our single-sex study design on experimental outcomes. Also, the method employed in our study to inhibit GAL3 was not specific, it did indeed promote the recovery of motor function in SCI. In the future, we plan to explore the effects of motor function recovery in both sexes by targeting the inhibition of GAL3 expression in specific spinal motor neurons.

In summary, our data reveal a close association between GAL3 and CDC42 with autophagy following SCI. In addition, GAL3 induces autophagy in spinal cord neurons, and GAL3 interacts with CDC42 after neuronal injury. Moreover, our study presents novel evidence that inhibitors TD139 and ML141 effectively reduce autophagy, contributing to functional recovery post-SCI. Our data indicate the therapeutic potential of targeting GAL3/CDC42 for enhancing recovery from spinal cord injuries.

## Data Availability

The datasets presented in this study can be found in online repositories. The names of the repository/repositories and accession number(s) can be found below: https://www.ncbi.nlm.nih.gov/, GSE274319.

## Footnotes

1
https://www.r-project.org/

2
https://bioconductor.org/packages/release/bioc/html/sva.html

3
https://cran.r-project.org/web/packages/limma/index.html

4
https://bioconductor.org/packages/release/bioc/html/clusterProfiler.html

5
https://ggplot2.tidyverse.org/

6
https://string-db.org/

7
http://www.cytoscape.org/
