# Supplementary material for: Galactin-3 regulation of CDC42 promotes neuronal autophagy following spinal cord injury
Source: Front Cell Neurosci. 2025 Oct 15;19:1622825. doi: 10.3389/fncel.2025.1622825 (PMC12568528; doi:10.3389/fncel.2025.1622825)
Supplement: Supplementary file 1 [file Supplementary_file_1.docx]

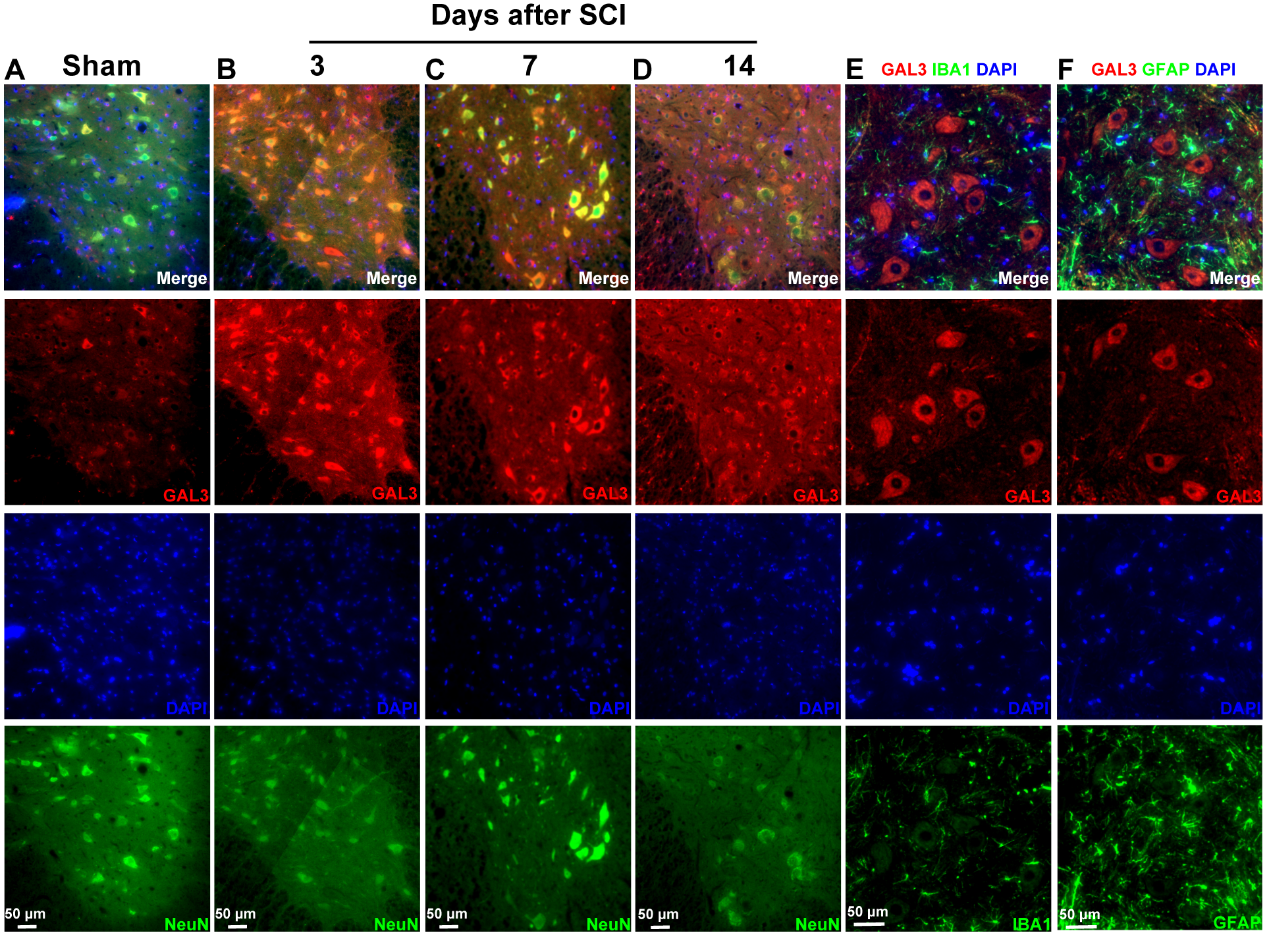


**Fig. S1** **Single-channel images of immunofluorescence microscopy in Figure 1**

**A,B,C,D** Immunofluorescence microscopy reveals GAL3 co-localization with NeuN sham-SCI or post-SCI. **E, F.** Immunofluorescence double staining of GAL3 and IBA1 (E) or GFAP (F) after SCI.


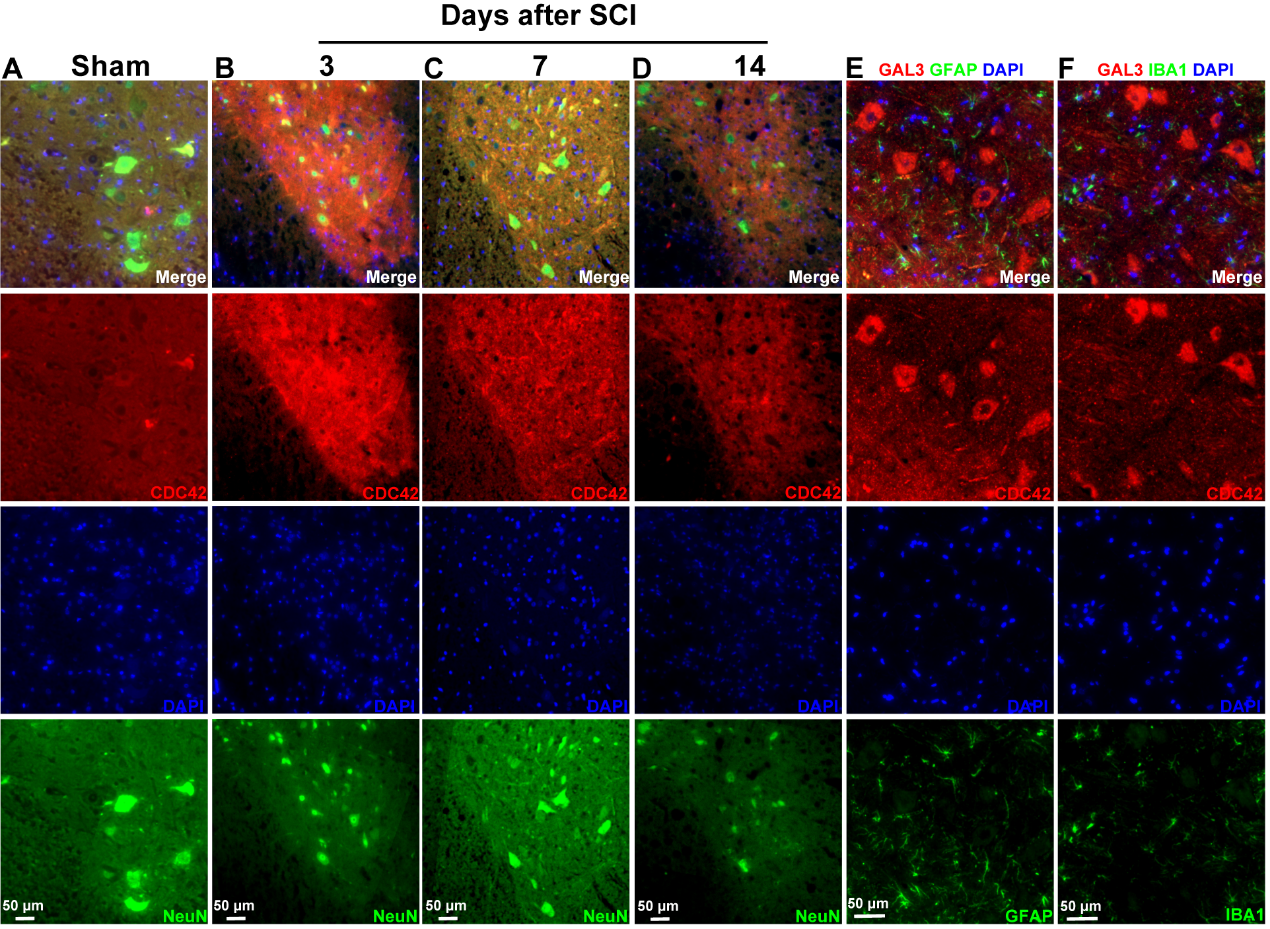


**Fig. S2** **Single-channel images of immunofluorescence microscopy in Figure 7**

**A,B,C,D** Immunofluorescence microscopy reveals CDC42 co-localization with NeuN sham-SCI or post-SCI. **E, F.** Immunofluorescence double staining of GAL3 and GFAP (E) or IBA1 (F) after SCI.

## 1．Sample collection and preparation

### 1.1 RNA isolation and qualification

RNA was extracted using the TRIzol method (Invitrogen, CA, USA) and treated with RNase-free DNase I (Takara, Kusatsu, Japan). RNA degradation and contamination was monitored on 1% agarose gels. RNA was quantified using Agilent 2100 Bioanalyzer (Agilent Technologies, CA, USA), the quality and integrity were assessed by NanoDrop spectrophotometer (Thermo Scientific, DE, USA).

### 1.2 Library preparation for Transcriptome sequencing

A total amount of 1.5 μg RNA per sample was used as input material for the RNA sample preparations. Sequencing libraries were generated using NEBNext® Ultra™ RNA Library Prep Kit for Illumina® (NEB, USA) following manufacturer’s recommendations and index codes were added to attribute sequences to each sample. Briefly, mRNA was purified from total RNA using poly-T oligo-attached magnetic beads. Fragmentation was carried out using divalent cations under elevated temperature in NEBNext First Strand Synthesis Reaction Buffer（5X）. First strand cDNA was synthesized using random hexamer primer and M-MuLV Reverse Transcriptase（RNase H）.

Second strand cDNA synthesis was subsequently performed using DNA Polymerase I and RNase H. Remaining overhangs were converted into blunt ends via exonuclease/polymerase activities. After adenylation of 3’ ends of DNA fragments, NEBNext Adaptor with hairpin loop structure were ligated to prepare for hybridization. In order to select cDNA fragments of preferentially 200-250 bp in length, the library fragments were purified with AMPure XP system (Beckman Coulter, Beverly, USA). Then 3 μl USER Enzyme (NEB, USA) was used with size-selected, adaptor-ligated cDNA at 37°C for 15 min followed by 5 min at 95°C before PCR. Then PCR was performed with Phusion High-Fidelity DNA polymerase, Universal PCR primers and Index (X) Primer. At last, PCR products were purified (AMPure XP system) and library quality was assessed on the Agilent Bioanalyzer 2100 system. The library preparations were sequenced on an Illumina Novaseq 6000 platform by the Beijing Allwegene Technology Company Limited (Beijing, China) and paired-end 150bp reads were generated.

## 2．Data analysis

### 2.1 Quality control

Raw data (raw reads) of fastq format were firstly processed through in-house perl scripts. In this step, clean data (clean reads) were obtained by removing reads containing adapter, reads containing ploy-N and low quality reads from raw data. At the same time, Q20, Q30, GC-content and sequence duplication level of the clean data were calculated. All the downstream analyses were based on clean data with high quality.

### 2.2 Mapping analysis

The adaptor sequences and low-quality sequence reads were removed from the data sets. Raw sequences were transformed into clean reads after data processing. These clean reads were then mapped to the reference genome sequence by STAR. Only reads with a perfect match or one mismatch were further analyzed and annotated based on the reference genome.

### 2.3 SNP calling

Picard - tools v1.41 and samtools v0.1.18 were used to sort, remove duplicated reads and merge the bam alignment results of each sample. GATK2 software was used to perform SNP calling. Raw vcffiles were filtered with GATK standard filter method and other parameters ( clusterWindowSize: 10; MQ0 >= 4 and (MQ0/(1.0*DP)) > 0.1; QUAL < 10; QUAL < 30.0 or QD < 5.0 or HRun > 5), and only SNPs with distance > 5 were retained.

### 2.4 Quantification of gene expression levels

HTSeq v 0.5.4 p3 was used to count the reads numbers mapped to each gene. Gene expression levels were estimated by fragments per kilobase of transcript per million fragments mapped (FPKM). The formula is shown as follow:


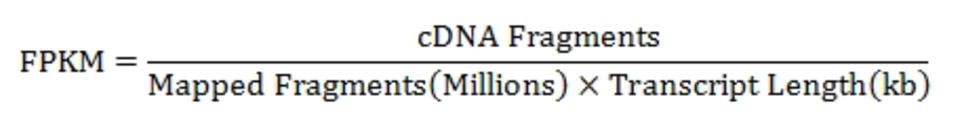


FPKM calculation formula

### 2.5 Differential expression analysis

**For the samples with biological replicates:**

Differential expression analysis of two conditions/groups was performed using the DESeq R package (1.10.1). DESeq provide statistical routines for determining differential expression in digital gene expression data using a model based on the negative binomial distribution. The resulting P values were adjusted using the Benjamini and Hochberg’s approach for controlling the false discovery rate. Genes with an adjusted P-value < 0.05 found by DESeq were assigned as differentially expressed.

**For the samples without biological replicates:**

Prior to differential gene expression analysis, for each sequenced library, the read counts were adjusted by edgeR program package through one scaling normalized factor. Differential expression analysis of two samples was performed using the DEGseq (2010) R package. Pvalue was adjusted using q value (Storey et al, 2003). qvalue < 0.005 & |log2（foldchange）| ≥1 was set as the threshold for significantly differential expression.

### 2.6 GO enrichment analysis

Gene Ontology (GO) enrichment analysis of the differentially expressed genes (DEGs) was implemented by the GOseq R packages based Wallenius non-central hyper-geometric distribution （Young et al, 2010），which can adjust for gene length bias in DEGs.

### 2.7 KEGG pathway enrichment analysis

KEGG (Kanehisa et al., 2008) is a database resource for understanding high-level functions and utilities of the biological system, such as the cell, the organism and the ecosystem, from molecular-level information, especially large-scale molecular datasets generated by genome sequencing and other high-throughput experimental technologies (http://www.genome.jp/kegg/). We used KOBAS (Mao et al., 2005) software to test the statistical enrichment of differential expression genes in KEGG pathways. **2.8 PPI (Protein Protein Interaction)**

The sequences of the DEGs was blast (blastx) to the genome of a related species (the protein protein interaction of which exists in the STRING database: http://string-db.org/) to get the predicted PPI of these DEGs. Then the PPI of these DEGs were visualized in Cytoscape (Shannon et al, 2005).

# References

1. Anders, S.(2010). HTSeq: Analysing high-throughput sequencing data with Python.(HTSeq)
2. Anders, S., and Huber, W. (2010). Differential expression analysis for sequence count data. Genome Biol.(DESeq)
3. Anders, S. and Huber, W. (2012). Differential expression of RNA-Seq data at the gene level-the DESeq package.(DESeq)
4. Kanehisa, M., M. Araki, et al. (2008). KEGG for linking genomes to life and the environment. Nucleic acids research.(KEGG)
5. Alexander Dobin, Carrie A.Davis, etal.(2013). STAR:ultra fast universal RNA-seqaligner.(STAR)
6. Langmead, B., Trapnell, C., Pop, M. & Salzberg, S.L. (2009). Ultrafast and memory-efficient alignment of short DNA sequences to the human genome. Genome Biol.(Bowtie)
7. Langmead, B. and S. L. Salzberg (2012). Fast gapped-read alignment with Bowtie 2. Nature methods.(Bowtie 2)
8. Mao, X., Cai, T., Olyarchuk, J.G., Wei, L. (2005). Automated genome annotation and pathway identification using the KEGG Orthology (KO) as a controlled vocabulary.
9. Bioinformatics.(KOBAS)
10. Marioni, J. C., C. E. Mason, et al. (2008). RNA-seq: an assessment of technical reproducibility and comparison with gene expression arrays. Genome research.
11. Marquez Y, Brown JW, Simpson C, Barta A, Kalyna M. (2012). Transcriptome survey reveals increased complexity of the alternative splicing landscape in Arabidopsis.
12. McKenna, A, Hanna, M, Banks, E, Sivachenko, A, Cibulskis, K, Kernytsky, A, Garimella, K, Altshuler, D, Gabriel, S, Daly, M, DePristo, MA. 2010. The Genome Analysis Toolkit: a
13. MapReduce framework for analyzing next-generation DNA sequencing data. Genome Research.(GATK)
14. Mortazavi, A., B. A. Williams, et al. (2008). Mapping and quantifying mammalian transcriptomes by RNA-Seq. Nature methods.
15. Robinson, M. D., McCarthy, D. J. & Smyth, G. K. edgeR: a Bioconductor package for
16. differential expression analysis of digital gene expression data. Bioinformatics.(edgeR)
17. Trapnell, C., Pachter, L., and Salzberg, S.L. (2009). TopHat: discovering splice junctions with RNA-Seq.
18. Trapnell, C. et al. (2010).Transcript assembly and quantification by RNA-seq reveals unannotated transcripts and isoform switching during cell differentiation. Nat.
19. Biotechnol.(Cufflinks)
20. Trapnell, C., A. Roberts, et al. (2012). Differential gene and transcript expression analysis of
21. RNA-seq experiments with TopHat and Cufflinks. Nature Protocols.(Tophat & Cufflinks)
22. Wang, L.Feng, Z.Wang, X.Zhang, X. (2010). DEGseq: an R package for identifying differentially expressed genes from RNA-seq data. Bioinformatics.(DEGseq)
23. Wang, Z., M. Gerstein, et al. (2009). RNA-Seq: a revolutionary tool for transcriptomics. Nature Reviews Genetics.
24. Young, M. D., Wakefield, M. J., Smyth, G. K., and Oshlack, A. (2010).Gene ontology analysis for RNA-seq: accounting for selection bias. Genome Biology.(GOseq)
25. Shihao Shen, Juw Won Park, et al. (2014). rMATS: Robust and flexible detection of differential alternative splicing from replicate RNA-Seq data.(rMATS)
